# Improvement of Pharmacokinetics Behavior of Apocynin by Nitrone Derivatization: Comparative Pharmacokinetics of Nitrone-Apocynin and its Parent Apocynin in Rats

**DOI:** 10.1371/journal.pone.0070189

**Published:** 2013-07-30

**Authors:** Kaiyu Wang, Linlin Li, Yan Song, Xiaocui Ye, Shaolian Fu, Jie Jiang, Sha Li

**Affiliations:** 1 Department of Pharmaceutics, College of Pharmacy, Jinan University, Guangzhou, The People’s Republic of China; 2 Institute of New Drug Research, College of Pharmacy, Jinan University, Guangzhou, The People’s Republic of China; University of Wuerzburg, Germany

## Abstract

Apocynin, a potent inhibitor of NADPH-oxidase, was widely studied for activities in diseases such as inflammation-mediated disorders, asthma and cardiovascular diseases. In our recent study, a novel nitrone derivative of apocynin, AN-1, demonstrated potent inhibition to oxidative injury and to high expression of gp91^phox^ subunit of NADPH-oxidase induced by tert-butyl hydroperoxide (t-BHP) in RAW 264.7 macrophage cells, and displayed promising preclinical protective effect against lipopolysaccharide (LPS)-induced acute lung injury in rats. In this work, the pharmacokinetic behaviors of AN-1 in Sprague-Dawley rats with single intravenous and intragastric doses were investigated for further development. Furthermore, apocynin’s pharmacokinetics remain lacking, even though its pharmacological action has been extensively evaluated. The pharmacokinetics of parent apocynin were also comparatively characterized. A simple HPLC method was developed and validated to determine both AN-1 and apocynin in rat plasma. The chromatographic separation was achieved on an Agilent HC-C18 column (250 mm×4.6 mm, 5 µm) at an isocratic flow rate of 1.0 mL/min, with the mobile phase of methanol and water (53∶47, v/v) and the UV detection set at 279 nm. Good linearity was established over the concentration range of 0.1–500 µg/mL for AN-1 and 0.2–100 µg/mL for apocynin. The absolute recovery, precision and accuracy were satisfactory. Compared with the parent compound apocynin, AN-1 yielded a much longer T_1/2_ (AN-1 179.8 min, apocynin 6.1 min) and higher AUC_0–t_ (AN-1 61.89 mmol/L·min, apocynin 2.49 mmol/L·min) after equimolar intravenous dosing (0.302 mmol/kg). The absolute bioavailability of oral AN-1 was 78%, but that of apocynin was only 2.8%. The significant improvement of pharmacokinetic behavior might be accounted for the effective pharmacodynamic results we documented for the novel nitrone derivative AN-1.

## Introduction

**Figure 1:**
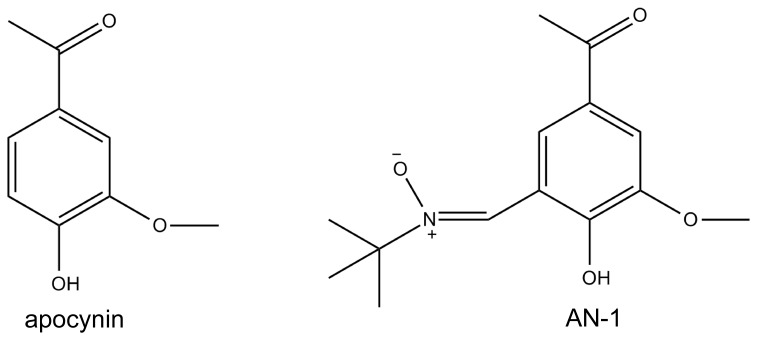
Chemical Structures of Apocynin and AN-1.

NADPH-oxidase is an enzyme responsible for reactive oxygen species (ROS) production. Excessive production of ROS would induce apoptotic cell death in various cell types, and deregulation of apoptosis causes clinical disorders. Thus, this enzyme was studied as an attractive therapeutic target for the treatment of many diseases [1], [2]. Apocynin (4-hydroxy-3-methoxyacetophenone, Figure 1) is an efficient inhibitor of NADPH-oxidase in a variety of cells and animal models [2]–[7]. It demonstrated potential therapeutic effect for many diseases attributed to NADPH-oxidase inhibition, including arthritis [3], [4], asthma [8], [9], stroke [6], [10], arteriosclerosis [11], and acute lung injury [12]–[16]. However, few studies about the structure modification of apocynin were carried out to explore more promising candidate entities. Recently, a series of new apocynin derivatives have been synthesized and evaluated for their biological activities in our lab [17], [18]. Results from previous studies [18] demonstrated that AN-1 ((Z)-N-(5-Acetyl-2-hydroxy- 3-methoxybenzylidene)-2-methylpropan-2-amine oxide, Figure 1), apocynin armed with a powerful free radical scavenging nitrone moiety, might also be a potent inhibitor of NADPH-oxidase by depressing the high expression of gp91^phox^ induced by t-BHP in RAW 264.7 macrophage cells. When compare to its parent chemical apocynin, AN-1 protected the RAW 264.7 cells against t-BHP inducing oxidative cytotoxicity as well as attenuated the ROS production markedly. In a rat model of LPS-induced acute lung injury, AN-1 showed a greater treatment effect than apocynin for the lung injury of model rats, and it significantly increased the activity of superoxide dismutase in the lung of model rats [18]. It suggested that AN-1 might be a potential candidate for the therapy of acute lung injury.

In drug discovery and development, a better understanding of the pharmacokinetics is very important for providing deep insight into the availability of drug development of a new chemical. The aim of our study was to determine the pharmacokinetics of apocynin and AN-1 after intravenous and oral administration in rats. In this study, a simple reliable HPLC-UV method was developed and validated for the determination of both AN-1 and apocynin in rat plasma, and then applied to the pharmacokinetic studies of single intravenous and intragastric doses, respectively, in Sprague-Dawley rats.

## Materials and Methods

### Chemicals and Reagents

AN-1 used in this study was synthesized in our lab by the same method as described previously [17]. The purity of AN-1 was higher than 99% confirmed by the HPLC analysis. Apocynin with a purity of 98% was obtained from Alfa Aesar Chemicals Co. Ltd (Tianjin, China). Carbamazepine used as internal standard (IS) was purchased from the Guangdong Institute for Drug Control (Guangdong, China). Methanol of chromatographic grade was purchased from Hanbon Scientific & Technology Co. Ltd (Jiangsu, China). Other chemicals and reagents were of analytical grade and commercially available.

### Instrumentation and Chromatographic Conditions

Analysis was carried out on an Agilent 1200 series HPLC system (Agilent Corporation, USA). The system consisted of a G1311A quaternary pump, a G1322A degasser, and a G1315B diode array detector. Chromatographic separation was achieved on an HC-C18 column (250 mm×4.6 mm, 5 µm, Agilent, USA) with a Security Guard column (10 mm×4.6 mm, 5 µm) filled with the same materials. The mobile phase consisted of a mixture of methanol-water (53∶47, v/v). Prior to use, the mobile phase was filtered through a 0.45 µm cellulosic Millipore membrane. The isocratic elution was run at a flow rate of 1 mL/min and the column was maintained at 30°C. The detection wavelength was 279 nm. The injection volume was 20 µL.

### Preparation of Standard and Quality Control Working Solutions

Standard stock solutions were prepared by dissolving AN-1 and apocynin in methanol at 5.0 mg/mL and 1.0 mg/mL, respectively. IS working solution was prepared by the same way at 0.8 mg/mL for AN-1 measurement and diluted to 40 µg/mL with methanol for apocynin measurement. A series of standard working solutions of AN-1 and apocynin, ranging from 1.0 to 5000 µg/mL and 1.0 to 500 µg/mL, respectively, were prepared by diluting the stock solution with methanol. Quality control (QC) working solutions of AN-1 (2, 2500 and 4000 µg/mL) and apocynin (2, 200 and 400 µg/mL) were prepared in the same way. All the solutions were stored at 4°C and brought to room temperature before use.

### Sample Preparation

The standard plasma samples for a calibration curve of AN-1 were prepared by spiking 10 µL of standard working solutions and 10 µL of IS solution (0.8 mg/mL) into 100 µL of drug-free blank plasma to achieve the final concentrations of AN-1 ranging from 0.1 to 500 µg/mL. After vortexing for 30 s, 180 µL methanol and 100 µL acetonitrile were added to the mixture to precipitate protein. Then the samples were vortex-mixed for 60 s and centrifuged at 14,000 rpm for 10 min at 4°C. The supernatant was injected into the HPLC system for analysis. The QC plasma samples for AN-1 with nominal concentrations of 0.2, 250, 400 µg/mL and the plasma samples obtained from pharmacokinetic study were prepared in the same way.

The samples of apocynin were prepared similarly, except that the volume of standard working solution and IS solution spiked were both changed to 20 µL, the concentration of IS solution was changed to 40 µg/mL, and the volume of methanol added to precipitate protein was changed to 60 µL. The concentrations of apocynin ranged from 0.2 to 100 µg/mL in the standard plasma samples. The QC plasma samples were set at 0.4, 40 and 80 µg/mL.

### HPLC Method Validations

#### Selectivity

The selectivity was assessed by comparing the chromatograms of blank plasma sample, spiked plasma samples, pre-dosing and post-dosing plasma samples obtained from pharmacokinetic study.

#### Linearity and lower limit of quantification (LLOQ)

A series of standard plasma samples were prepared with standard working solutions as described above. The peak areas of analytes and IS were recorded to calculate peak area ratios. The calibration curves were constructed by least squares linear regression of the peak area ratios versus the corresponding concentrations with a weighing factor of 1/x^2^. The calibration curve was prepared daily. The LLOQ was defined as the lowest concentration on the calibration curve, at which the signal-to-noise (S/N) ratio was not less than 10, with precision and accuracy within 20% and 80%–120%, respectively.

#### Precision and accuracy

The QC plasma samples of three concentration levels were evaluated for intra-day and inter-day precision and accuracy of AN-1 and apocynin, five replicates for each concentration and testing for five consecutive days. The precision was expressed as a percentage of relative standard deviation (RSD%). RSD% was calculated according to the formula: 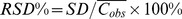, where the 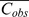 is the average observed concentrations of QC plasma samples. The accuracy was calculated by comparing the observed concentration (C_obs_) with the nominal concentration (C_nom_ ) of analytes in QC plasma samples according to the formula: Accuracy (%) = C_obs/_C_nom_×100%.

#### Recovery and stability

The recovery was evaluated by using a set of replicates (n = 5) at three QC concentrations. The recovery was calculated by comparing the peak areas of analytes in the spiked QC plasma samples with the average peak area of analytes in authentic solutions of the same concentration. The recovery of IS was measured in the same way.

The stability of analytes in rat plasma stored under different conditions was assessed at low, medium and high QC concentrations, respectively. The conditions included long-term storage at −20°C for 30 days, short-term storage at room temperature (8 h for AN-1, 24 h for apocynin), three freeze-thaw cycles treatment, and post-preparative QC samples stored at room temperature (8 h for AN-1, 24 h for apocynin). The concentrations measured from the above samples were compared with the nominal values of QC plasma samples. Samples were concluded stable and applicable to routine analysis if the deviation of the observed concentration was less than ±15% from the nominal value.

### Animals

Sprague-Dawley rats were purchased from Guangdong Medical Laboratory Animal Center (Guangdong, China). The animals were housed in an environmentally controlled breeding room with free access to standard laboratory food and water. All animals were kept 7 days for acclimation before experiments. All the animal studies described in this paper were approved and conducted in accordance with the guidelines of Laboratory Animal Ethics Committee of Jinan University (Approval ID: 20120308001).

### Pharmacokinetic Study

Male Sprague-Dawley rats, weighing 210–290 g, were fasted for 12 h but with free access to water before dosing. The rats were randomly divided into groups of intravenous AN-1, intravenous apocynin, intragastric AN-1 and intragastric apocynin. AN-1 was dissolved in a mixture of PEG400-ethanol-saline (5∶2∶3, v/v) due to its poor solubility in water for administration. Apocynin was dissolved in a mixture of propylene glycol-saline (4∶6, v/v). The single-dose pharmacokinetic study was performed. For AN-1, three different doses (20, 40 and 80 mg/kg) were administered for intravenous study (n = 6–8) and one dose (40 mg/kg) was given for intragastric study (n = 6). For apocynin, 50 mg/kg, an equimolar dose to 80 mg/kg (0.302 mmol/kg) of AN-1, was administered for both intravenous (n = 8) and intragastric studies (n = 6). A day before dosing, a cannula was introduced into jugular vein on rats for intravenous dosing and blood sampling in all groups.

Blood samples (0.4 mL) was collected via jugular vein into heparinized tubes at 0 min (before dosing) and 5, 10, 15, 30, 45, 60, 90, 120, 180, 240, 360, 480, 600, 720, 840 min for AN-1 and at 0 min (before dosing) and 2, 4, 6, 10, 15, 20, 30, 40, 50, 60 min for apocynin after intravenous and intragastric dosing. An equal volume of heparinized saline was used to flush the catheter immediately after each blood withdrawal to prevent blood clotting. The blood samples were centrifuged immediately at 10,000 rpm for 5 min at 4°C, and then the plasma was transferred and stored at −20°C until analysis.

The profiles of analyte concentrations in rat plasma versus time were analyzed to calculate pharmacokinetic parameters by a non-compartmental model using DAS software (version 3.0.1, Chinese Mathematical Pharmacology Society).

## Results and Discussion

### Development of HPLC Method

Several methods for determination of apocynin and some nitrone derivatives in biological samples have been reported [19]–[22]. Both HPLC and LC/MS were used for measurements. The analytes were generally separated by gradient elution with complicated mobile phase systems and long analysis time, or by isocratic elution with buffer salt in the mobile phase. Based on the reported studies, we chose the HPLC method for its general availability and optimized chromatographic conditions for the resolution and quantification of AN-1 and apocynin in rat plasma. Different compositions of the mobile phase (methanol/acetonitrile and water/buffer solution) were tested. We observed that a simple mobile phase consisting of methanol-water at a ratio of 53∶47 (v/v) without adding any buffer system yielded a satisfactory peak shape, separation and peak height response within 14 min. The maximum absorption wavelengths of AN-1 and apocynin were 279 nm and 276 nm, respectively. The wavelength of 279 nm was chosen for the quantification. In this study, carbamazepine was selected as the IS because of its high recovery and suitable retention time. The retention time of apocynin, AN-1 and IS were approximately 4.8 min, 7.1 min and 11.3 min, respectively.

Regarding to the relatively complicated and time-consuming liquid-liquid extraction, the simple procedure of protein precipitation was used for the plasma sample preparation. Different protein precipitation solvents, such as methanol and/or acetonitrile were tested. Finally, methanol-acetonitrile mixture (2∶1 for AN-1 and 1∶1 for apocynin, v/v) was adopted with a complete protein precipitation of high recovery and good repeatability.

### HPLC Method Validations

#### Selectivity

**Figure 2:**
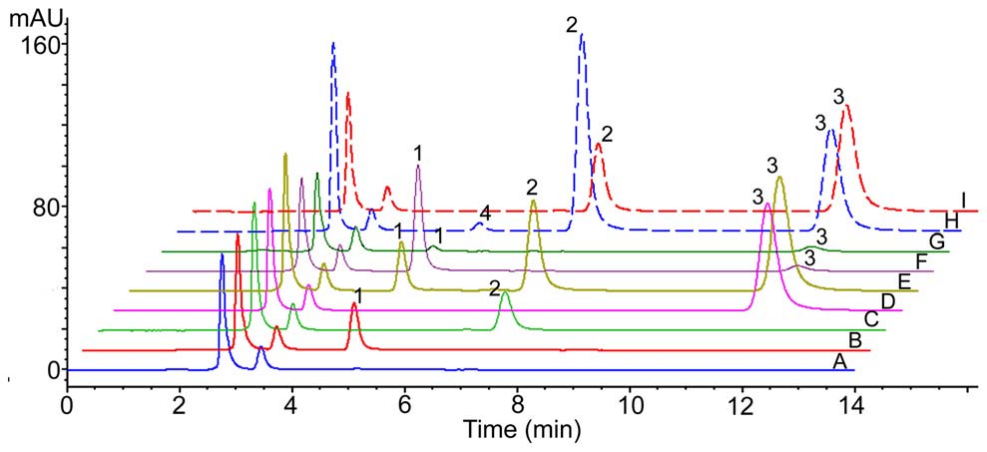
Typical Chromatograms for Selectivity of HPLC-UV Method. Chromatograms of (A) blank plasma; (B) blank plasma spiked with apocynin (10 µg/mL); (C) blank plasma spiked with AN-1 (8 µg/mL); (D) blank plasma spiked with IS (80 µg/mL); (E) blank plasma spiked with apocynin (10 µg/mL), AN-1 (20 µg/mL) and IS (80 µg/mL); (F) plasma sample collected at 6 min after intravenous injection of apocynin (50 mg/kg) spiked with IS (8 µg/mL); (G) plasma sample collected at 4 min after intragastric administration of apocynin (50 mg/kg) spiked with IS (8 µg/mL): (H) plasma sample collected at 1 h after intravenous injection of AN-1 (80 mg/kg) spiked with IS (80 µg/mL); (I) plasma sample collected at 30 min after intragastric administration of AN-1 (40 mg/kg) spiked with IS (80 µg/mL). Peaks of 1, apocynin; 2, AN-1; 3, IS (carbamazepine); 4, proposed metabolite.

Typical chromatograms of blank plasma, blank plasma spiked with apocynin, AN-1 and IS, and rat plasma samples collected after the administration of apocynin or AN-1 are shown in Figure 2. The chromatograms of pre-dosing plasma samples were same as that of blank plasma. No endogenous interferences were observed at the retention times of apocynin, AN-1 and IS in any of the biological samples, indicating the high selectivity of this analysis for the analytes in plasma matrix.

#### Linearity and LLOQ

The calibration curves with good linearity were established over the concentration range of 0.1–500 µg/mL for AN-1 and 0.2–100 µg/mL for apocynin. The mean equation of the calibration curves of AN-1 generated on five different days was 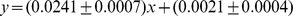, and that of apocynin was 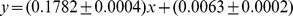, where *y* was the ratio of peak areas of analyte to IS and *x* was the concentration of analyte in each standard plasma sample. All the linear correlation coefficients (r^2^) were above 0.999 and the accuracies of the calibration standards were acceptable. The LLOQ of AN-1 was 0.1 µg/mL with the precision less than 8% and the mean accuracy of 92.7%. The LLOQ of apocynin was 0.2 µg/mL with the precision of 1.1% and the mean accuracy of 91.5% (Table 1).

**Table 1:** Precision and Accuracy for the Determination of AN-1 and Apocynin in Rat Plasma (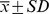, n = 5).

Analytes	Concentration added	Precision (RSD %)		Accuracy (%)
	(µg/mL)	Intra-day	Inter-day	
AN-1	0.1^a^	7.88	–	92.69±7.30
	0.2	2.70	3.83	100.08±2.70
	250	0.27	1.49	98.77±0.27
	400	4.46	1.12	97.82±4.36
Apocynin	0.2^a^	1.11	–	91.54±1.02
	0.4	1.51	2.98	101.54±1.53
	40	2.10	2.11	92.06±1.94
	80	2.64	1.44	94.08±2.48

a n = 6.

#### Precision and accuracy

The intra- and inter-day precision and accuracy of AN-1 and apocynin are listed in Table 1. The intra- and inter-day precision of 3 QC concentrations of AN-1, expressed as RSD (%), ranged from 0.27 to 4.46%. That of apocynin was within 1.44 to 2.98%. The accuracy of QC samples of AN-1 and apocynin was ranged from 97.8% to 100.1% and 92.1 to 101.5%, respectively. The values were within the acceptable criteria of precision less than 15% and accuracy within 85%–115%.

#### Recovery and stability

The recovery of AN-1, apocynin and IS are summarized in Table 2. The mean recoveries of AN-1 and apocynin were higher than 84.4% and 94.4%, respectively at all three concentrations of QC samples. The mean recoveries of IS were higher than 91.2%. The relative standard deviations for the recovery were less than 3.87%, 3.94% and 3.61% for AN-1, apocynin and IS respectively, indicating the good consistency.

**Table 2:** Extraction Recoveries of AN-1, Apocynin and IS in Rat Plasma (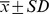, n = 5).

Analytes	Concentration added	Extraction recovery	RSD
	(µg/mL)	(%)	(%)
AN-1	0.1^a^	85.77±2.25	2.62
	0.2	84.39±3.27	3.87
	250	90.45±0.77	0.85
	400	90.26±0.86	0.95
Apocynin	0.2^a^	93.43±1.82	1.95
	0.4	97.81±3.85	3.94
	40	94.39±2.25	2.38
	80	100.31±2.04	2.03
IS	8	96.77±3.49	3.61
	80	91.18±0.62	0.68

a n = 6.

As shown in Table 3, AN-1 and apocynin were stable after a long-term storage at −20°C, short-term storage at room temperature and complete three freeze-thaw cycles, with all of the accuracy higher than 90%. The post-preparative solutions of both AN-1 and apocynin were also stable at room temperature for at least 8 h. The results indicated that the plasma samples stored in the freezer immediately after the blood sampling and analyzed within 30 days offered reliable results.

**Table 3:** Accuracy of AN-1 and Apocynin in Rat Plasma in Stability Test under Different Conditions (%, 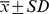, n = 5).

Conditions	AN-1 Concentration (µg/mL)			Apocynin Concentration (µg/mL)		
	0.2	250	400	0.4	40	80
Long-term stability	92.98±1.91	94.74±2.43	91.92±0.86	102.17±0.87	95.18±0.34	94.09±0.99
Short-term stability	89.96±0.31	91.99±1.37	91.46±0.96	102.47±6.10	95.52±1.01	94.11±1.35
Freeze-thaw stability	94.79±0.46	95.56±0.95	96.33±0.98	102.87±1.62	95.48±0.69	93.60±1.79
Post-preparative stability	88.61±0.58	88.14±0.84	91.96±1.51	98.34±6.26	94.57±0.60	92.51±1.43

### Pharmacokinetic Study and Data Analysis

**Figure 3:**
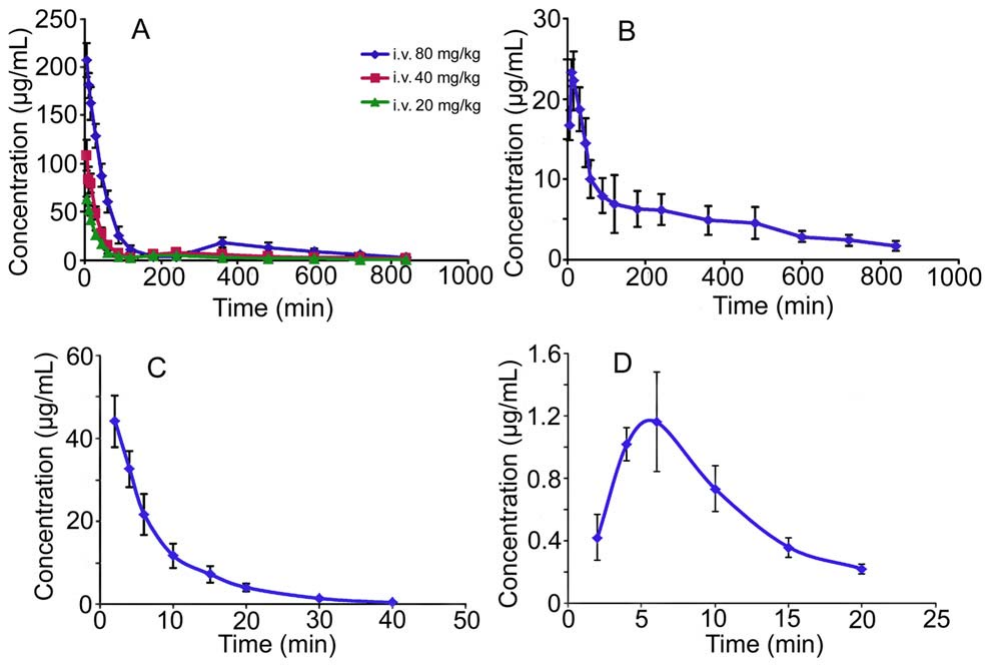
Mean Plasma Concentration-Time Profiles of AN-1 and Apocynin in Sprague-Dawley Rats. Profiles of (A) AN-1 after a single intravenous dose at 20 mg/kg (n = 8), 40 mg/kg (n = 6) and 80 mg/kg (n = 8); (B) AN-1 after a single intragastric dose at 40 mg/kg (n = 6); (C) apocynin after a single intravenous dose at 50 mg/kg (n = 8); (D) apocynin after a single intragastric dose at 50 mg/kg (n = 6). Each point represented as mean ± SD.

**Figure 4:**
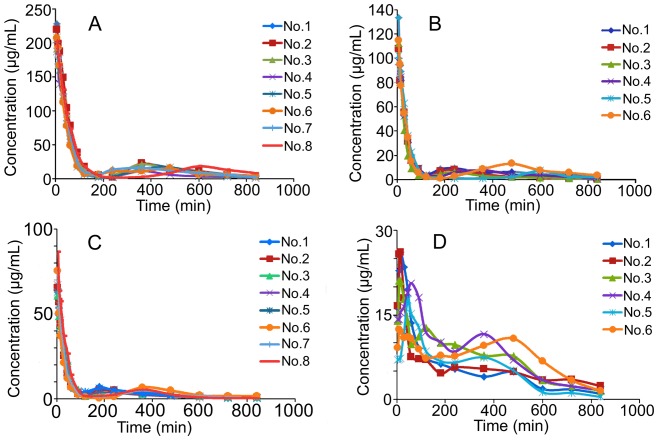
Individual Plasma Concentration-time Profiles of AN-1 in Sprague-Dawley Rats. Profiles of (A) AN-1 after a single intravenous dose at 80 mg/kg (n = 8); (B) AN-1 after a single intravenous dose at 40 mg/kg (n = 6); (C) AN-1 after a single intravenous dose at 20 mg/kg (n = 8); (D) AN-1 after a single intragastric dose at 40 mg/kg (n = 6).

**Figure 5:**
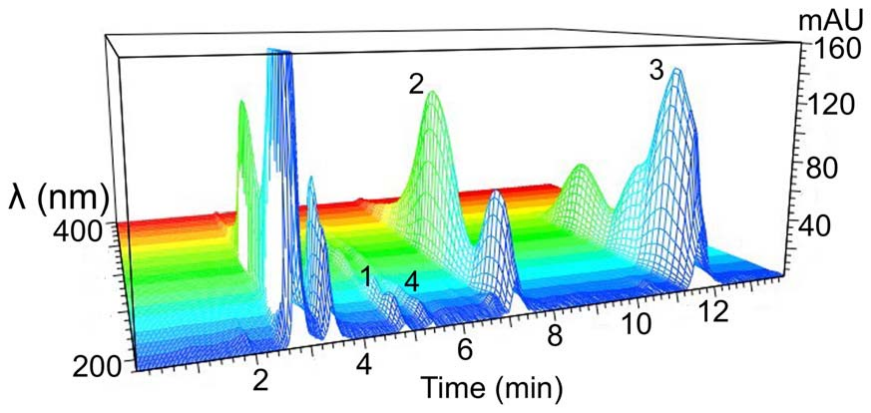
Representative Chromatogram of Plasma Sample Collected at 1 h after Intravenous Injection of AN-1 (80 mg/kg) Containing the Unknown Metabolite Spiked with IS (80 µg/mL) and Apocynin (10 µg/mL). Peaks of 1, apocynin; 2, AN-1; 3, IS (carbamazepine); 4, proposed metabolite.

The validated HPLC assay was successfully applied to the pharmacokinetic studies of AN-1 and apocynin in Sprague-Dawley rats. The mean plasma concentration-time profiles after intravenous and intragastric administrations are illustrated in Figure 3. The main pharmacokinetic parameters analyzed by non-compartmental model are listed in Table 4. The AN-1 plasma concentration declined rapidly over the first 2 h (120 min) after intravenous administration and then increased slightly over the 3 to 8 h (180–480 min) time period (Figure 3A and 4A–C). After intragastric dosing, AN-1 was absorbed quickly (T_max_ = 25.8±21.3 min) to reach a C_max_ of 0.08±0.02 mmol/L. The plasma level then dropped quickly followed by a slight increase over the 2 to 8 h (120–480 min) time period. However, due to the great individual difference of the second plasma concentration peak time, the phenomenon was only shown in the profiles of each rat but not in the mean concentration-time profile (Figure 3B and 4D). The double peak phenomenon indicated the possible presence of enterohepatic recycling of AN-1 in rats. A metabolite of AN-1 was detected in the plasma from 5 min after intravenous administrations at 40 mg/kg and 80 mg/kg, the retention time was about 5.2 min. The metabolite was not apocynin as confirmed by the chromatogram of pharmacokinetic plasma sample of AN-1 containing metabolite spiked with apocynin (Figure 5). The concentration of apocynin dropped significantly and rapidly within 40 min in both intravenous and intragastric concentration-time profiles (Figure 3C–D). The T_max_ and C_max_ were 5.3±1.0 min and 0.008±0.002 mmol/L, respectively. No double peak phenomenon was observed for apocynin in any of the studied rats.

**Table 4:** Main Pharmacokinetic Parameters of AN-1 and Apocynin in Sprague-Dawley Rats (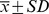).

Parameters	Units	Apocynin			AN-1				
		Intravenous	Intragastric	F	Intravenous			Intragastric	F
		50 mg/kg^a^	50 mg/kg^b^	(%)	20 mg/kg^a^	40 mg/kg^b^	80 mg/kg^a^	40 mg/kg^b^	(%)
AUC_0–-t_	mmol/L·min	2.49±0.46	0.07±0.01	2.8	14.88±1.31	26.51±5.12	61.89±7.59	20.68±3.95	78.0
MRT_0–t_	min	7.0±0.8	8.1±0.7		176.1±34.5	193.0±53.8	205.1±26.1	290.9±39.3	
T_1/2_	min	6.1±0.9	5.9±1.8		171.7±79.9	162.6±56.3	179.8±44.9	201.1±97.0	
T_max_	min	–	5.3±1.0		–	–	–	25.8±21.3	
C_max_	mmol/L	–	0.008±0.002		–	–	–	0.08±0.02	
V	L/kg	1.09±0.30	31.46±12.14		1.20±0.54	1.27±0.32	1.20±0.32	1.95±0.90	
CL	L/min/kg	0.12±0.02	3.67±0.35		0.005±0.001	0.006±0.001	0.005±0.001	0.007±0.001	

a n = 8,

b n = 6.

The pharmacokinetic parameters of AN-1 of the three intravenous dosage groups were analyzed statistically by ANOVA analysis. The T_1/2_, clearance (CL) and apparent volume of distribution (V) had no significant difference (P>0.05) among different dosage groups. The AUC_0–t_ showed good linear relationship with the doses (r^2^ = 0.9913), suggesting the dose linearity over the 20–80 mg/kg range after intravenous dosing.

As shown in Table 4, AN-1 possessed a significantly longer terminal elimination half-life (162.6–201.1 min) than that of apocynin (5.9–6.1 min). After receiving an equimolar intravenous dose (0.302 mmol/kg), the exposure of AN-1 in the systemic circulation (AUC_0–t_ 61.89±7.59 mmol/L·min) was 25-fold of that of apocynin (AUC_0–t_ 2.49±0.46 mmol/L·min). The potential enterohepatic recycling of AN-1 might be one of the key factors which resulted in the long terminal elimination half-life and high AUC_0–t_ of AN-1. The long T_1/2_ and high AUC_0–t_ might contribute significantly to the better therapeutic efficacy of AN-1 than apocynin in an acute lung injury rat model [18]. The AUC_0–t_ for intragastric administration of AN-1 (40 mg/kg) was 20.68±3.95 mmol/L·min, and the absolute oral bioavailability was 78%. However, the absolute oral bioavailability of apocynin was negligible as 2.8%. It is generally believed that solubility and membrane permeability are the key determinants of oral bioavailability of a given compound [23]. AN-1 and apocynin were both given in solution form, the solubility barrier no longer existed here. The difference in oral bioavailability between AN-1 and apocynin might be partly attributed to their different membrane permeability which needs further investigation. The fast elimination, short half-life and low oral bioavailability may limit the use of apocynin. The marked improvement of pharmacokinetic characteristics after the nitrone derivatization of apocynin offered a prospective feasibility for the further development of AN-1.

### Conclusions

In conclusion, a simple and reliable HPLC method was developed and fully validated for quantifications of AN-1 and apocynin in rat plasma. The method could be used for future biopharmaceutical and organ distribution studies. The pharmacokinetic behaviors of both AN-1 and apocynin were comparatively characterized in Sprague-Dawley rats, to better understand their kinetic characteristics *in vivo*. A bimodal phenomenon was observed in concentration-time profiles of AN-1, however, the enterohepatic recycling still needs further confirmation and characterization. The metabolite of AN-1 in rat plasma will also be studied in the future. The AN-1 had a longer elimination half-life, higher AUC and oral bioavailability than its parent compound apocynin. The results were significant in potentially correlating to the observed potent action of AN-1 in the acute lung injury rat model, and to offer rational strategy for the future development of AN-1.
